# Calling for a re‐evaluation of the data required to credibly demonstrate a dental student is safe and ready to practice

**DOI:** 10.1111/eje.12191

**Published:** 2016-03-29

**Authors:** L. J. Dawson, B. G. Mason, V. Bissell, C. Youngson

**Affiliations:** ^1^ University of Liverpool School of DentistryLiverpoolUK; ^2^ University of Glasgow School of DentistryGlasgowUK

## Introduction

In the UK, the Francis Report 1 has driven key changes within health care and has focused the need to:

‘Make all those who provide care for patients – properly accountable for what they do… to ensure that the public is protected from those not fit to provide such a service.’ 1

Irrespective of nationality, this statement underscores the importance of undergraduate education, and its associated assessments, because the best and most cost‐effective way to protect the public is to ensure that only the right individuals go on the professional register in the first place. For providers of undergraduate education, this distils down into the problem of how to ensure and demonstrate that our graduates are competent to practice.

In undergraduate dental education, common approaches for demonstration of competency are grounded in the traditions of novice to expert learning 2. In this arena, becoming an ‘expert’ requires ten or more years of experience 3. Consequently, the traditional method for determining competency is the measurement of experience through counting the number and the quality of procedures completed 4. This approach has likely become widely accepted because it appears to have face validity; it is simple to do; progression decisions can easily be defended; it has endured the test of time; and it fulfils a crucial criterion for assessment, namely it is acceptable to stakeholders 5. Data to support the latter statement can be found through reference to the latest round of inspection reports by the UK General Dental Council (GDC) where a focus on, and a drive to increase, the numbers of individual procedures performed by undergraduate learners is still very evident 6.

However, is this traditional approach still the best possible way of measuring competency considering the aforementioned changes in expectation over accountability, combined with advances in our understanding of pedagogy, and available technology?

This paper aims to initiate debate over what should constitute best practice in the assessment of competence. From the evidence‐base available we suggest that to truly establish competency sophisticated approaches for data collection, integration, and interpretation are likely to be needed to meet the demands and expectations of the 21st century. This is because the modern healthcare setting requires its professionals to be responsive and adapt to the ever‐changing needs of patients 7. We suggest that in this setting, the important evidence underpinning competency is the longitudinal demonstration of the learner's ability to independently and simultaneously manage all aspects of the activity being assessed for each patient, over a range of contexts, rather than simply measuring the amount of a specific activity or isolated facets of competency such as communication or professionalism. Furthermore, we will contend that decisions over progression will need to be made on a leaner‐specific basis through the professional judgement, and consensus of a multidisciplinary expert panel following the objective analysis of large and fully integrated data sets.

## What is professional competence?

Professional competence has been defined as:

‘The habitual and judicious use of communication, knowledge, technical skills, clinical reasoning, emotions, values, and reflection in daily practice for the benefit of the individual and community being served.’ 8

Therefore, the assessment of professional competence is complicated because it requires the daily integration of all data to demonstrate the stability and appropriateness of multiple skills and behaviours over time (habitual*)*. This complex situation is often managed by assimilating the various dimensions of professional competence into a series of outcomes, which are then further organised into a series of domains such as clinical, communication, professionalism and management and leadership. This is the situation that exists in the UK and is described by the GDC in the document ‘Preparing for Practice’ 9.

The ability to convincingly establish competence in each domain is a fundamental requirement for defensible decisions over student progress or graduate registration. Therefore, establishing a suitable approach for the measurement of competence warrants careful consideration.

In 1998, David Chambers published a landmark paper entitled *Competency‐based dental education in context* 2. In this publication, he explored the available data in the spectrum of novice‐expert learning. He noted that five distinct developmental stages were recognised, novice, beginner, competent, proficient and expert, and that becoming an expert requires ten or more years of experience 3. He concluded that *five years is only enough time to make a good start* 2 and that graduates would, at best, only be at the level of competent. In this paradigm, ‘competent’ refers to a developmental stage that is:

‘Marked by independence, supported by basic internalized standards and an acceptable repertoire of skills and knowledge.’ 2

Moving forward, the components that describe the developmental stage of ‘competent’, such as independence, internalised standards, appropriate repertoire of skills and knowledge and understanding, would seem suitable to inform a guiding framework for exploring the measurement of competency at the domain level.

## What are the limitations of current approaches to measuring competence?

In dentistry, competency has traditionally been measured through establishing the levels of activity (i.e. numbers of procedures) 4. However, data from multiple studies in medicine support the conclusion that experience does not necessarily predict competency 10, 11, 12, and in some cases may be associated with a reduction in competency 11. Moreover, in dentistry, at least one small study suggests that there is no significant difference in outcome between beginners and experts when the focus is on the end product:

‘The traditional evaluation criteria in dental education (numbers of tasks completed or their quality defined in objective terms) are probably insufficient to reliably distinguish the level of learning of emerging professionals.’ 4

Whilst there can be no doubt that increased levels of activity broaden experience, it will become evident that it is the type of activity that is important and not the amount of activity that improves competence. The difference in focus between activity type and amount is likely to be decisive because an emphasis on the quantity can lead to learners concentrating on completing tasks, ultimately seeing patients as commodities that are only useful whilst their care contributes to the required skills tally. This is likely to have a deleterious educational impact 13. This is because it is a situation that can only detract from students actively pursuing patient‐centred holistic treatment and gaining the required integrated learning approaches.

To facilitate a more robust measurement of competency, we would suggest a move away from the idea of progression through the developmental stages being solely driven by experience, towards the driver being *enhancing performance through deliberate practice* 14. We recognise that many schools are well aware of the limitations of a purely quantitative approach and have already made adjustments to their assessment strategy.

## What is the relationship of competence to performance?

The concept of novice to expert learning, within a construct of performance, has been investigated and eloquently described by Anders Ericsson as:

‘Nobody becomes an outstanding professional without experience, but extensive experience does not invariably lead people to become experts’…. Although everyone in a given domain tends to improve with experience initially, some develop faster than others and continue to improve during the ensuing years. These individuals are eventually recognised as experts and masters. In contrast, most professionals reach a stable, average level of performance within a relatively short time frame and maintain this mediocre status for the rest of their careers.’ 14

Ericsson 14, 15 proposed a model to explain how *professionals reach a stable performance asymptote within a limited time period, whereas the expert performers are able to keep improving their performance for years and decades*.

When a learner is first introduced to a new activity, their primary goal is to reach a level in that domain which is deemed to be acceptable. At this early stage, the learner needs to concentrate hard to avoid mistakes. With more appropriate and focused practice directed by feedback, combined with domain‐specific experience, the performance becomes smoother and requires less concentration until eventually it becomes automated. At the stage of automation, the individual loses conscious awareness and therefore is no longer able to make *specific intentional adjustments* without additional external observation and feedback 14. This concept of automation is decisive because once a professional has reached an acceptable skill level, data suggest that more experience does not lead to improved performance 15. Experts, on the other hand, continually and deliberately seek the continued training situation designed to place the desired goal beyond their current level of achievement.

The goal of undergraduate education may not be to create experts but it can certainly utilise the concept of deliberate practice in the development of learners. However, this requires the creation of a powerful learning environment 16 in which a number of key components will need to be brought together:

- Learners are systematically challenged through increasing task difficulty to prevent ‘automation’.
- Teachers continually monitor learner performance.
- The provision of multisource feedback from both staff and patients, which is appropriately detailed and timely 17 to enable reflection and subsequent performance modification through *deliberate (focused) practice* 14, 15.
- Continuous opportunities and encouragement for the learner to undertake deliberate practice.

Success in such an environment is predicated around the ability to appropriately measure performance. Without this ability, both the meaningful monitoring of performance and the capability of providing the required levels of feedback become impossible.

## How can performance be measured?

In assessment, established wisdom is that any measurement must be in relation to some form of transparently applied criteria or standard. Data from a recent study suggest that measurement scales that are constructively aligned 18 to the level of expertise of the assessor and the developing independence of the learner reduce the levels of disagreement between assessors and thus improve confidence in the assessment outcome 19. Furthermore, the measurement of performance through developing independence is also entirely consistent with the aforementioned required components of competence.

We suggest that a numerical scale anchored to descriptors over the degree of *independence* and *quality* of the learner's performance represents a justifiable approach for measurement that also drives the appropriate educational impact. In addition, with the right longitudinal and triangulated approach, it is a method that could not only be used to inform both the quality and consistency of domain‐specific skills, but, by direct inference, also be used to inform both the quality and consistency of the internalised standards being applied by the learner, as well as measuring the learner response to external feedback through analysis of the degree of subsequent change in performance.

The need to assess daily practice, whilst at the same time capturing performance in multiple contexts, implicates the use of Workplace Based Assessment tools (WBAs), either in current or modified forms, linked to the aforementioned numerical scale. WBAs have been shown to have good predicative reliability 20, 21. However, data suggest that great care has to be used in the way they are operationalised and used to make decisions 19, 22. Some of the big challenges are as follows: (i) in the real world, patients, tasks and situations are subject to huge variability, (ii) WBAs are traditionally carried out on a limited number of occasions and designed for a specific task, which gives the learner a task rather than holistic focus and (iii) WBAs are subject to decisions from staff that will be influenced by the context in which they are made and the individuals who are making them. Amongst other things, these issues have resulted in the realisation that *from a psychometric perspective, very large numbers of assessors and cases are required to discriminate reproducibly amongst trainees* 22; and the need for a change in both terminology and focus when considering the qualitative data from WBAs. It is also of note that it has been suggested that the terms ‘credibility’ (cf. internal validity) and ‘dependability’ (cf. reliability*)* 23 better describe the aims for the trustworthiness of type of data collected through this approach.

Irrespective of the terminology used, for WBAs to be employed successfully in the determination of competence, there would seem to be an implicit requirement for the collection, integration and active interpretation, of large continuous and longitudinal data sets. This is because without them, it would not be possible to establish the pattern of different performances across many different contexts within or across the domain(s) of interest.

## How often should performance be measured and in what contexts?

Having established a principle for performance measurement, it is necessary to consider the available evidence to inform how often and where such measurements should take place. In other words, what is the acceptable repertoire of skills, what is an appropriate breadth of patients/procedures and what is a sufficient number of occasions to develop the skills?

Medical education is dominated by constructivist views of learning that consider learning as an ‘entity’ where the context within which the learning occurs may affect its quality, but has little impact on the ‘learning’ itself 24. A direct consequence of this conventional view is that competence is regarded as a trait, which once achieved is stable irrespective of context. This implies that for any individual skill, the degree of competence can be established, and once acquired is directly transferable to any situation that arises requiring that skill.

However, data strongly suggest that competence is highly context specific 12, 25. Furthermore, modern health care requires its professionals to be responsive to the needs of patients. The ability of an individual to respond to required change has been described as ‘capability’, which is defined as the *extent to which an individual can adapt to change, generate new knowledge, and continue to improve their performance* 7. Taken together, this means *that we can no longer see competence as ‘a state to be achieved’… Competence is not just about acquisition of knowledge and skills, but about the ability to create new knowledge in response to changing work processes* 24. This paradigm shift means that *modern healthcare systems demand that we assess our learners ability to adapt and to flexibly apply and develop knowledge* 24.

Placing these arguments into the arena of dentistry, we would suggest that the true assessment of competence requires a demonstration of the learner's consistency in their ability to simultaneously integrate and appropriately apply and adapt all the relevant domain‐specific skills at the required level of independence, across a range of contexts. Thus, the breadth of experience becomes at least as important as amount. For instance, in Restorative Dentistry, the breadth of context for the placement of direct restorations might include tooth surface, tooth location, material used, difficulty of task (e.g. access, extent of caries and medical history), patient demography (age, gender, ethnicity, disability, anxiety level, etc.) and environment (clinical discipline, in‐reach, outreach, etc.). With this approach, the amount of data required to demonstrate competency will be large, variable and learner specific. This is because each learner will see different patients, each contributing a specific set of contexts. Furthermore, each leaner will develop at their own rate and will likely have different deliberate practice and feedback needs to stabilise their longitudinal performance.

## Knowledge and understanding

The final facet of domain‐associated competency to consider is knowledge and understanding. The principles for the objective measurement of knowledge 5, 26, 27, and the appropriate formats within which to do it, are well established and there is no need to elaborate upon them further here.

However, just as with any other component of competence, it cannot be assumed that once the student assimilates knowledge that they will have sufficient understanding to apply it when they come across any relevant situation. This is highlighted by data from a study evaluating the influences of teaching on learning that quote the response of a medical student support this view:

‘I found it very difficult to actually study something like ‘head injury’ without relating it to my own personal knowledge of the clinical situation….. I think it is ludicrous to teach something like ‘head injury’…. without having that clinical basis, because (then) you (remember) what you are learning as a series of disconnected facts… (just) a very efficient way to pass an exam.’ 28

Long‐standing data from work exploring child development provide insight into the problem, as it suggests that the ability to apply knowledge is also highly contextual and requires experience to allow the knowledge to be consolidated and organised in an appropriate way 29, 30, 31. This need for knowledge transformation through experience has been well established in medical education 32, 33 and has greatly influenced those studying the development of diagnostic expertise 34, especially in the area of clinical reasoning skills 25, 35.

Overall, data support the hypothesis that the acquisition of knowledge should be developed and concurrently monitored alongside the relevant clinical exposure in real time across contexts of skills application.

## The triangulation and aggregation problem

The arguments presented suggest that the data needed for a true demonstration of professional competence are large and complex, with an implicit need for a coherent approach to aggregation and triangulation. Multiple assessment types designed and considered in isolation may lack the required sophistication, a situation that would be true irrespective of how well the various pieces of data were blueprinted together, or how valid and reliable 26 each of the individual assessments were considered to be. To illustrate, a situation that will be familiar to dental academics is one where a student causes concern to experienced clinical faculty. However, the student is pleasant, has managed to undertake the requisite amount of experience and has passed the available WBA's, OSCE's and knowledge examinations. There is probably good cause for the staff concern, but the student's progression is assured because the available data, although spanning domains, are considered in self‐contained ‘assessment packets’, that is they are barely passing in several areas but the outcome is nevertheless a pass. It should be considered that someone in this situation is probably not competent overall, but the available data and the way it is integrated lack sufficient sophistication and resolution to reflect the legitimate concerns of the experienced teachers. We would contend that in the situation of a dental programme, a true measure of competency cannot be established from isolated assessments even when they are triangulated together, be they OSCEs, WBAs or written tests, especially where the focus of aggregation is the assessment instrument rather than the domain or skill. We propose that an enhanced measurement process that ensures the right outcomes for learners, patients and stakeholders is required. It is a process underpinned by the full integration and triangulation of data from all domains and contexts combined with an understanding of the performance within them. Crucially, within this paradigm, data from simulation, objective assessment and patients should be viewed as different contexts, which through appropriate assessment design strategies involving a coherent approach can be integrated to demonstrate competence. Clearly, it will be necessary to identify where triangulation is appropriate, and work in postgraduate medicine developing ‘Entrustable Professional Activities’ where data are required to be integrated and triangulated from many competencies spanning multiple domains to holistically demonstrate a real‐world skill, may be a good model 36. If clinical academics were able to evaluate each and every clinical episode in terms of a cross‐domain data set, where any outcome falling below the required level of independence highlighted an insufficiency, then this would allow them to reflect the overall ability of the learner to holistically manage the patient on that occasion. The data derived from individual episodes of patient treatment would be integrated on a longitudinal basis and interpreted to determine patterns of consistency, which when further triangulated across contexts and with other assessment data would give a closer reflection of the true competency of the learner. Further benefits of such an approach is that it could (i) with the right management of WBA data allow for identification and moderation of staff who were not giving the learners appropriate feedback, in essence ‘failing to fail’, a know issue in dentistry 37 and (ii) serve to enhance the utility of assessment because its purpose is not just to identify who passes or fails, but rather to make the assessment process part of everyday learning and reflection 38.

Crucially, the aggregation, triangulation and interpretation of this personalised and complex data derived from a variety of contexts will not be straightforward or, lend itself to a purely quantitative approach. This will necessitate a move from individual disciplines behaving as independent entities when making progress decisions towards an integrated approach where a multidisciplinary panel functions as an *interpretive community* 24 to establish the ability of an individual to practise dentistry because:

‘“Truth” is a matter of consensus among assessors who have to arrive at judgments on performance that are as informed and sophisticated as can be at a particular point in time’ 24.

## Recommendations for data to inform a decision over competency

Overall, the available data strongly suggest that the demonstration of competence requires *a* coherent approach to the longitudinal aggregation and triangulation of data. Based on our analysis of the available evidence, the following five broad principles are suggested to inform credible, dependable and trustworthy decisions over learner progression:

- Consistency, demonstrated through the longitudinal measurement of performance is a key parameter to establish competence. Measurement of performance should be grounded in the developing independence of the learner.
- Both the *breadth* (i.e. the different contexts) and the consistency (number of *occasions* at the appropriate level) of performance are key drivers in developing and demonstrating competence. A number of parameters in relation to each assessed performance should be recorded as an indicator of context and enable the triangulation of data between and across contexts.
- In a dental programme, single assessments are not the best way of establishing or developing student competence, as these do not provide a sufficient breadth of contexts, an appropriate educational impact or longitudinal insight. Sophisticated methods of assessment data collection, integration and triangulation both within and across domains are required.
- It is essential that knowledge be linked to real‐world patient encounters in multiple contexts, as well as from appropriately aligned theoretical and simulated situations that require the learner to process information and make relevant clinical decisions in a highly aligned and contextual manner.
- Progress decisions are best reached through the judgement of a multidisciplinary interpretive community informed by comprehensive data and a sophisticated approach to interpretation as discussed.
